# Bioprospecting of Natural Compounds from Brazilian Cerrado Biome Plants in Human Cervical Cancer Cell Lines

**DOI:** 10.3390/ijms22073383

**Published:** 2021-03-25

**Authors:** Marcela N. Rosa, Larissa R. V. e Silva, Giovanna B. Longato, Adriane F. Evangelista, Izabela N. F. Gomes, Ana Laura V. Alves, Bruno G. de Oliveira, Fernanda E. Pinto, Wanderson Romão, Allisson R. de Rezende, Arali A. C. Araújo, Lohanna S. F. M. Oliveira, Alessandra A. de M. Souza, Stephanie C. Oliveira, Rosy Iara M. de A. Ribeiro, Viviane A. O. Silva, Rui M. Reis

**Affiliations:** 1 Molecular Oncology Research Center, Barretos Cancer Hospital, Barretos 14784-400, Brazil; nr.marcela2@gmail.com (M.N.R.); larirussoveloso@yahoo.com.br (L.R.V.eS.); giovanna.longato@usf.edu.br (G.B.L.); adriane.feijo@gmail.com (A.F.E.); izabela.faria.tk@hotmail.com (I.N.F.G.); alves.anav@gmail.com (A.L.V.A.); vivianeaos@gmail.com (V.A.O.S.); 2 Research Laboratory in Molecular Pharmacology and Bioactive Compounds, São Francisco University, Bragança Paulista 12916-900, Brazil; 3 Petroleomic and Forensic Laboratory, Chemistry Department, Federal University of Espírito Santo, Vitória 29075-910, Brazil; brunoliveir_ra20@msn.com (B.G.d.O.); fernandapinto80@gmail.com (F.E.P.); wandersonromao@gmail.com (W.R.); 4 Higher Institute of Education and Research of Ituiutaba, University of the State of Minas Gerais (UEMG), Ituiutaba 38302-192, Brazil; rodrigues.allisson@gmail.com (A.R.d.R.); arali.dacov@gmail.com (A.A.C.A.); 5 Laboratory of Experimental Pathology, Federal University of São João del Rei—CCO/UFSJ, Divinópolis 35501-296, Brazil; lohh.franca@gmail.com (L.S.F.M.O.); aleapms@gmail.com (A.A.d.M.S.); stephaniecorsino.ufsj@gmail.com (S.C.O.); rosy@ufsj.edu.br (R.I.M.d.A.R.); 6 Life and Health Sciences Research Institute (ICVS), School of Medicine, University of Minho, 4710-057 Braga, Portugal; 7 ICVS/3B’s-PT Government Associate Laboratory, 4710-057 Braga, Portugal

**Keywords:** cervical cancer, cytotoxicity, Annonaceae, Melastomataceae, Fabaceae, Asteraceae

## Abstract

Cervical cancer is the third most common in Brazilian women. The chemotherapy used for the treatment of this disease can cause many side effects; then, to overcome this problem, new treatment options are necessary. Natural compounds represent one of the most promising sources for the development of new drugs. In this study, 13 different species of 6 families from the Brazilian Cerrado vegetation biome were screened against human cervical cancer cell lines (CCC). Some of these species were also evaluated in one normal keratinocyte cell line (HaCaT). The effect of crude extracts on cell viability was evaluated by a colorimetric method (MTS assay). Extracts from *Annona crassiflora*, *Miconia albicans*, *Miconia chamissois*, *Stryphnodendron adstringens*, *Tapirira guianensis*, *Xylopia aromatica*, and *Achyrocline alata* showed half-maximal inhibitory concentration (IC_50_) values < 30 μg/mL for at least one CCC. *A. crassiflora* and *S. adstringens* extracts were selective for CCC. Mass spectrometry (Electrospray Ionization Fourier Transform Ion Cyclotron Resonance Mass Spectrometer (ESI FT-ICR MS)) of *A. crassiflora* identified fatty acids and flavonols as secondary compounds. One of the *A. crassiflora* fractions, 7C24 (from chloroform partition), increased H2AX phosphorylation (suggesting DNA damage), PARP cleavage, and cell cycle arrest in CCC. Kaempferol-3-O-rhamnoside and oleic acid were bioactive molecules identified in 7C24 fraction. These findings emphasize the importance of investigating bioactive molecules from natural sources for developing new anti-cancer drugs.

## 1. Introduction

Among several cancer types with a higher incidence in Brazil, cancer of the uterine cervix is the third most common in women, excluding non-melanoma skin cancer (NMSC) [1]. In 2018, around 570,000 new cases were estimated, with a mortality of 311,000 women worldwide [2]. In Brazil, mortality in women from this type of cancer follows world estimates, representing the fourth leading cause of cancer death, excluding NMSC [3]. The drugs used in chemotherapy treatment are platinum-based. However, this treatment can cause many side effects and it has a limited influence on survival in patients with advanced or recurrent cancer [4,5]. New therapeutic options are needed to overcome these problems.

Natural compounds represent one of the most promising sources for new drug discovery. From 1940 to 2014, 175 small molecules were approved for cancer treatment, including 131 (75%) of non-synthetic origin and 85 (49%) being natural products or their derivatives [6]. The chemotherapeutics vincristine, vinblastine, paclitaxel, and podophyllotoxin are examples of antitumor compounds of plant origin. By the end of 2020, about 127 clinical trials were being performed with plant-derived molecules such as l-selenomethionine (phases II and III), napabucasin (phases I/II, II and III), genistein (phases I, II and I/II), idronoxil (phases I, II and I/II), and gossypol (phases I/II and II) in different cancer types, according a search in the Clarivate Analytics Integrity database. Brazil has the highest biodiversity on the planet [4,7,8]; however, despite the amount of scientific studies with Brazilian plant species, in-depth phytochemical studies are still scarce [7,9,10]. The natural Brazilian landscape is distributed throughout its six biomes: Amazon, Caatinga, Cerrado, Atlantic Forest, Pampa, and Pantanal [11], with Cerrado representing the second largest biome, with a vast diversity of natural plants [12]. Because of the dry climate with seasonal rain, this biome presents characteristic upland vegetation on deep and well-drained soils [13]. The trees, shrubs, and herbaceous plants have characteristics unique to their adaptation, such as the production of secondary metabolites that protect against herbivory and pathogens [14,15].

These metabolites also have beneficial effects on human health. For this reason, some Cerrado plants are traditionally used to treat issues such as bacterial, fungal, or parasitic infections, gastrointestinal disorders, arthritis, respiratory disorders, wound healing, and cancer-related conditions [13,16,17].

We evaluated the in vitro effect of different Brazilian Cerrado plant species against cervical cancer, which has a high incidence rate in Brazil. For this, 13 crude extracts from the leaves of plants from 6 families (Fabaceae, Anacardiaceae, Annonaceae, Siparunaceae, Asteraceae, and Melastomataceae) were screened in human cervical cancer cell (CCC) lines. *Annona crassiflora* was selected for the partitioning process, using four different solvents with increasing polarity: alcohol, hexane, chloroform, and ethyl acetate. The chloroform partition of *A. crassiflora* was fractionated, and the 7C24 fraction was evaluated for proteins related to DNA damage, apoptosis, and cell cycle regulation.

Our findings identified compounds in *A. crassiflora* with biological activity and revealed cytotoxic potential in extracts from Cerrado vegetation that can contribute to the development of new anti-cancer drugs.

## 2. Results

**Figure 1:**
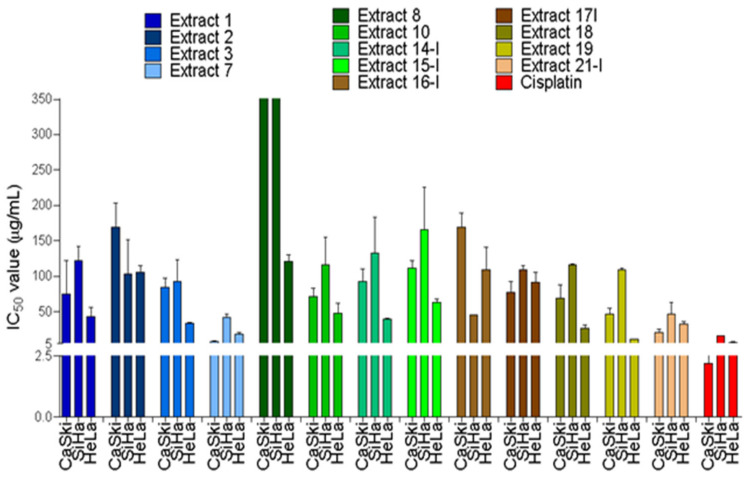
Effect of different natural crude extracts on viability of cervical cancer cell lines. Cellular viability was measured at 72 h by MTS assay. The results were expressed as the mean percentage ± SD of viable cells relatively to the DMSO alone (viability considered to be 100%). The half-maximal inhibitory concentration (IC_50_) concentrations were calculated by nonlinear regression analysis using Graphpad Prism software. Data represent the mean of at least two independent experiments performed in triplicate. Concentrations of crude extracts ranged from 2.5 to 300 µg/mL. Cisplatin concentrations ranged from 0.12 to 30 µg/mL.

After 72 h treatment, screens revealed half-maximal inhibitory concentration (IC_50_) values ranging from 8.24 to 169.50 μg/mL, when all extracts were considered for all cancer cell types in the main panel (Figure 1 and Table 1). It was not possible to determine an IC_50_ value for some cell lines, as the highest concentration tested was not sufficient to inhibit cell viability by 50% (Figure 1 and Table 1).

The National Cancer Institute’s screening program indicates that crude extracts yielding IC_50_ values < 30 µg/mL are candidates for purification (Suffness and Pezzuto, 1990 *apud* De Mesquita). In this study, four species showed IC_50_ values < 30 μg/mL in at least one of the lines in the central panel: *Annona crassiflora* (7), *Miconia albicans* (18), *Miconia chamissois* (19), and *Stryphnodendron adstringens* (21-I). Three species showed lower IC_50_ values in the cancer cell lines listed in Table S1 (Supplementary Materials): *Tapirira guianensis* (1), *Xylopia aromatica* (3), and *Achyrocline alata* (10).

The species that presented the lowest IC_50_ values in all main panel cell lines were *Annona crassiflora* (7) (which ranged from 8.24 to 42.44 μg/mL) and *Stryphnodendron adstringens* (21-I) (from 21.12 to 47.01 μg/mL).

We next evaluated the selectivity index (SI) for these species based on the response (IC_50_ value) of a normal human skin keratinocyte cell line, HaCaT. HaCat cell line is commonly used to represent in vitro human papillomavirus (HPV) infection-induced carcinogenesis [18]. The analysis showed selectivity (SI higher than two) for *A. crassiflora* and *S. adstringens* in CaSki and HeLa cell lines (Table 2). Based on these results, we selected extract 7 (*A. crassiflora*) to proceed with the partitioning process).

Cisplatin, a chemotherapeutic used in clinical practice, showed IC_50_ values of 2.21 to 15.48 μg/mL in CCCs (Figure 1 and Table 1).

The IC_50_ values ranged from 7.03 to 37.21 μg/mL for all compounds and all cell lines in the main panel (Table 3). It was not possible to determine the IC_50_ value of 7A for SiHa, because the highest concentration tested was not sufficient to inhibit cell viability by 50%. The effect of 7B partition on CCC viability was reported in Silva et al. (2018) [19].

Electrospray Ionization Fourier Transform Ion Cyclotron Resonance (ESI FT-ICR) mass spectrometry was performed to characterize the constituents of the natural compounds. The profile of the *A. crassiflora* chloroform partition indicated the presence of fatty acids and flavonols (Table 4). The constituents of the hexane partition are described elsewhere [19]. The m/z values of the main molecules found in the *A. crassiflora* chloroform partition are shown in Table 4.

Partition 7C was identified as one of the most cytotoxic for CCCs and was chosen for additional viability assays and evaluation to identify the possible mechanism of action.

Comparing the fraction activity on cell viability, fraction 7C24 yielded IC_50_ values lower than 30 µg/mL in HeLa and C4-I cell lines; 7C25 in C4-I; 7C28 in CaSki, HeLa and C4-I; 7C57 in C4-I; and 7C60 in HeLa and C4-I (Table 5). Fraction 7C45 had effect in HeLa and C4-I, with IC_50_ slightly higher than 30 µg/mL. Fractions 7C18, 7C22, 7C39, and 7C52 had no effect at any tested concentration and were not evaluated further (Table 5).

Among the fractions that reduced viability in all cell lines, 7C24 had one of the lowest IC_50′_s and was selected for further study. We selected resistant SiHa and sensitive HeLa cell lines to investigate the effect of 7C24 in processes such as cell death. SiHa and HeLa cells were treated with their respective IC_50_ values of 7C24 (33.88 and 15.96 µg/mL, respectively) and cisplatin (13.01 and 9.36 µg/mL, respectively). After 24 h, Western blotting showed histone H2AX phosphorylation and PARP cleavage in SiHa, and an increase in these events in HeLa cells, suggesting 7C24 modulated the DNA damage and cell death pathways.

**Figure 2:**
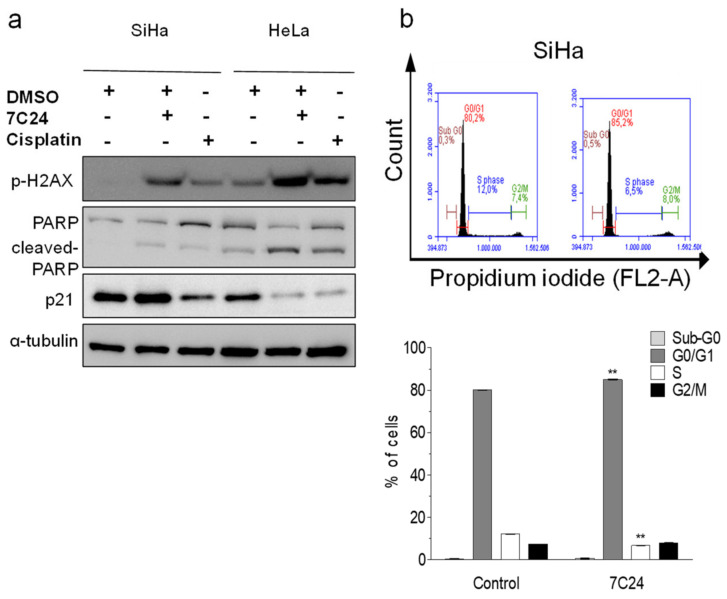
Fraction 7C24 and cell processes (DNA damage, apoptosis, and cell cycle) in SiHa and HeLa cell lines. SiHa and HeLa cells were treated with 7C24 (IC_50_ 33.88 and 15.96 µg/mL, respectively) and cisplatin (13.01 and 9.36 µg/mL, respectively). Western blotting for phospho-H2AX, PARP, and p21 after 24 h of treatment (**a**). Flow cytometry of SiHa 24 h after 7C24 treatment, mean ± SD of two independent experiments (**b**). C + (present),—(absent). ** *p* < 0.01.

The 7C24 fraction induced a slight increase in p21 expression in SiHa cells and a decrease in HeLa cells. Cisplatin induced a slight increase in PARP cleavage and H2AX activity and decreased p21 expression in both cell lines (Figure 2a). Changes in p21 can indicate cell cycle modulation. To investigate this, we used flow cytometry to evaluate the effect of 7C24 fractions on the SiHa cell cycle and observed an increase in G0/G1 phase-arrested cells (80.1 ± 0.10 to 85.0 ± 0.29%) and a reduction in S phase (from 12.1 ± 0.18 to 6.7 ± 0.25%) (Figure 2b).

ESI FT-ICR mass spectrometry showed the chloroform fraction of 7C24 contained oleic acid and kaempferol-3-O-rhamnoside (Table 6).

## 3. Discussion

Brazilian flora represents a vast resource for the discovery of substances with biological properties, and studies have shown that extracts from a variety of plants have cytotoxic effects on human tumor cell lines [10,19,25,26]. In this work, we investigated the cytotoxic effect of 13 plants from 6 families found in the Brazilian Cerrado biome on cervical human cancer cell lines. From these plants, we found IC_50_ values lower than 30 µg/mL after treatment with *Annona crassiflora*, *Miconia albicans*, *Miconia chamissois*, *Stryphnodendron adstringens*, *Tapirira guianensis*, *Xylopia aromatica* and *Achyrocline alata*. *A. crassiflora* and *S. adstringens* were tested and showed selectivity for cancer cells. The bioassay-guided fractionation of *A. crassiflora* revealed the effect of one fraction, 7C24, on pathways in DNA damage and apoptosis, and caused cell cycle arrest.

The Annonaceae family includes 135 genera and 2500 species distributed mainly in tropical zones [27]. *Annona* is the second largest genus of this family, corresponding to approximately 166 species [28]. Among *Annona* species, in vitro antitumor potential has been demonstrated for many compounds isolated from the essential oils of leaves of *A. vepretorum* and *A. sylvatica* [28]. Different parts of *A. squamosa*, *A. muricata*, *A. coriacea*, and *A. cacans* plants carry acetogenin compounds which exhibit activity against human cancer cell lines [29,30]. These acetogenin compounds are typically found in *Annona sp.*, and they are well known as potent inhibitors of mitochondrial respiratory chain complex I and nicotinamide adenine dinucleotide (NADH) oxidase [28].

Extracts and compounds isolated from *A. crassiflora* possess antitumor properties (methanolic and hexane extract), antimalarial effects (acetogenins, alkaloids, and flavonoids enriched fractions), antimicrobial and antioxidant activities (flavonoids), and chemopreventive and anti-inflammatory effects (methanolic extract) [19]. Formagio et al. (2015) reported the potential of methanolic extract from the leaves of the plant in 10 human cancer cell lines. After 48 h, the concentrations able to inhibit the growth of 50% of cells (GI_50_) ranged from 2.49 to 44.83 µg/mL, and only one cell line was resistant to the highest tested concentration. In the same study, the crude extracts from seeds were even more promising, with GI_50_ values of 0.01 to 8.90 µg/mL [31]. Mesquita et al. (2009) also showed this characteristic in root bark extracts, with IC_50_ values of 6.00 to 14.90 µg/mL in colon and brain cancer, melanoma, and leukemia cell lines [16]. We recently explored the antitumor activity of this extract and its hexane partition on cervical cancer, revealing that both compounds reduced the number of colonies in the clonogenic assay and induced alterations indicating DNA damage and apoptosis by intrinsic pathway [19].

In this study, we showed that the chloroform fractions reduced cell viability. One of these fractions, 7C24, induced alterations suggestive of DNA damage and apoptosis (H2AX phosphorylation, PARP cleavage) and modulated the cell cycle, observed as changes in p21 expression and arrest in the G0/G1 phase in SiHa cells. NMR analysis of the 7C24 fraction revealed different molecules as its constituents. Among them we identified the kaempferol-3-O-rhamnoside and oleic acid. Although it is not possible to affirm the effect is due to those molecules, the role of each one of them was already described in the literature. Kaempferol-3-O-rhamnoside is a glycosyloxyflavone identified in the Indonesian plant, *Schima wallichii (S. wallichii) Korth*. (Theaceae family). The compound has shown promising results in the induction of apoptosis in breast and prostate cancer [32,33]. Moreover, this same compound attached to an alpha-L-rhamnosyl residue, named afzelin, was isolated from *Nymphaea odorata* and exhibited anti-cancer activity against androgen-sensitive LNCaP and androgen-independent PC-3 prostate cancer cells through the inhibition of LIM domain kinase 1, which catalyzes various processes in cancer progression [34].

Oleic acid is a fatty acid already found in seed from *Annona hypoglauca*, from where it presented activities against diseases other than cancer [35]. Regarding the effect on cancer cell proliferation, the role of oleic acid is controversial [36]. In general, the anticancer effect of oleic acid has been evaluated mainly in the context of the constitution of olive oil in the “Mediterranean diet,” showing the ability to promote apoptosis (by increasing intracellular reactive oxygen species production or caspase 3 activity) [36], and reducing autophagy [37].

During the initial screening of natural extracts, the cytotoxic potential can provide a guide to isolating fractions purer than the crude extract, which, in general, are expected to be more bioactive [38]. Despite fraction 7C24 presenting fewer molecules in NMR analysis than the chloroform partition, the increased IC_50_ did not confirm a higher cytotoxic potential than partition. This could be explained by characteristics of secondary metabolites, which can interact, producing synergistic, additive, or antagonistic effects [39].

Beyond *A. crassiflora*, it is noteworthy that the other twelve species tested presented some degree of cytotoxic potential. During the initial screening of natural extracts, this potential can provide a guide to isolating fractions purer than the crude extract, which, in general, are expected to be more bioactive [38]. Interestingly, one of the species with a high IC_50_ value, *Bauhinia variegata*, when submitted to fractionation cycles by our group, reduced viability of MDA-MB231 human breast cell line after 24 h, exhibiting an IC_50_ value of 10.2 µg/mL [40]. This fraction was selective for tumor cells and inhibited the mechanisms of breast cancer metastasis, preventing spread to the lung and inflammation in the liver [40]. The constituents identified in this fraction, obtained by ethyl acetate partition, suggested that fatty acids and flavonoids are correlated with such activities [40].

We also evaluated *M. chamissois* and showed the effect of the crude extract on cervical cancer cell viability, but this effect was more evident in other cell types, such as breast (data not published) and glioblastoma [41]. In glioblastoma cell lines, the chloroform partition reduced colony formation, cell migration, and invasion and induced intrinsic apoptosis [41]. Furthermore, one flavonoid identified after fractioning, matteucinol, showed the same effect and reduced tumor growth and angiogenesis in the in vivo chick chorioallantoic membrane assay [41], leading to the exploitation of a new treatment for glioblastoma.

Some species that produced extracts with the ability to inhibit cervical cancer cell viability have been tested in other tumor types. Recently, a leaf fraction of *Stryphnodendron adstringens* containing gallic acid, procyanidin dimer B1, and (−)-epicatechin-3-O-gallate showed antioxidant activity and cytotoxicity toward human breast cancer cell lines [42]. A proanthocyanidin polymer-rich fraction from stem bark promoted in vitro and in vivo cancer cell death via oxidative stress in the cervical cancer model [43].

Ethanolic extracts from the aerial parts and flowers of *Achyrocline alata* are cytotoxic to human hepatocellular carcinoma cell lines but not normal primary human hepatocytes [44].

Our article represents the first report of the cytotoxic activity of crude leaf extracts of *Tapira guainensis* and *Xylopia aromatica*. Woody tissue extracts have been evaluated elsewhere and showed cytotoxic effects at 100 µg/mL against a central nervous system cancer cell line SF-268 (*T. guainensis*) and a leukemia cancer cell line RPMI-8226 (*X. aromatica*) [45]. This report is also the first to describe the activity of crude *Astronium fraxinifolium* extract on cancer cells.

Only one report has described the antitumor potential of leaf extracts from *Siparuna guianensis*, which exhibited cytostatic activity against human and mouse cancer cells, including breast cancer [46].

In this study, most screened extracts reduced viability of human cancer cell lines and is the first time that this cytotoxic potential has been reported for some species. Through bioactivity-guided fractionation cycles, some putative bioactive compounds were identified in *A. crassiflora*, with fraction 7C24 promoting cell cycle arrest, DNA damage, and cleavage of PARP in cervical cancer cells. These findings contribute to the investigation of bioactive molecules from natural sources and their potential in developing new therapies for cervical cancer.

## 4. Materials and Methods

### 4.1. Plant Material and Crude Extracts

Thirteen plant species from the Brazilian Cerrado biome were collected in Ituiutaba, MG (18°58′08″ S; 49°27′54″ W) or Uberlândia-MG (18°54′24.53″ S; 48°13′53.79″ W), between November 2007 and 2008. After identification, they were deposited in the Herbarium of the Federal University of Uberlândia (HUFU), the Herbarium of the Federal University of Minas Gerais (BHCB), or the Herbarium of the Federal University of Mato Grosso do Sul, Campo Grande campus, Brazil (CGMS), as indicated in Table 7.

The leaves were washed, air-dried and ground into a powder. Then, 100 g of each specie were extracted in an hydroethanolic solution (7:3), with a ratio of powder plant material/solvent of 1:5. The extracts were filtered and freeze-dried to obtain a crude extract and different yield were obtained, as follows: specie 1 (2.1%), 7 (approximately 10%), 8 (13.2%), 10 (2.5%), 14-I (4.5%), 15-I (6.9%), 16-I (3.7%), species 2, 3, 17-I, 18 and 19 (approximately 15%) and 21-I (17%). Screens were performed with extracts 1, 2, 3, 7, 8, 10, 14-I, 15-I, 16-I, 17, 18, 19, and 21-I (Table 7). They were diluted in dimethylsulfoxide (DMSO) to 50 mg/mL and stored at −20 °C. The concentrations tested were 6.25; 12.5; 25; 50; 100; 200 and 300 μg/mL.

### 4.2. Bioactivity-Guided Fractionation

After screening the crude extracts, the most cytotoxic, *Annona crassiflora* (7), was selected for partitioning. This partitioning, already described in Silva et al. [19], was performed using four different solvents with increasing polarity: alcohol, hexane, chloroform and ethyl acetate, partitions identified as A, B, C, and D. The partitions were diluted in DMSO to 25 mg/mL and stored at −20 °C. The concentrations tested were 0.78, 1.56, 3.13, 6.25, 12.5, 25, and 50 μg/mL. The concentration of DMSO never reached more than 1%.

Once the chloroform partition was identified as one of the most cytotoxic for cervical cancer cells, it was fractionated by classical chromatography, resulting in several fractions. All the fractions with enough material available were tested, ten in total, identified as 7C18, 7C22, 7C24, 7C25, 7C28, 7C39, 7C45, 7C52, 7C57, and 7C60.

### 4.3. Characterization of Secondary Compounds by Mass Spectrometry (ESI FT-ICR MS)

To identify the molecules present in the chloroform partition and the fraction 7C24 of *A. crassiflora*, they were submitted for analysis in a negative ion-mode Electrospray Ionization Fourier Transform Ion Cyclotron Resonance Mass Spectrometer (ESI (−) FT-ICR MS, model 9.4 T Solarix, Bruker Daltonics). The mass spectra were externally calibrated using NaTFA, with the m/z from 200 to 2000). The parameters of the ESI (−) source were: capillary voltage 3–3.5 kV, capillary transfer temperature 250 °C and nebulizer gas pressure of 0.5–1.0 bar. The resolution power was m/Δm_50%_ ≅ 200,000 (where Δm_50%_ is the maximum peak width at peak height m/z ≅ 400) and mass accuracy < 8 ppm. The equation double bond equivalent (DBE) = c − h/2 + n/2 + 1 (where c, h, and n are the numbers of carbon atoms, hydrogens, and nitrogen in the molecular formula, respectively) was used to deduce the degree of unsaturation for each molecule directly from its DBE value. The FT-ICR mass spectrum was acquired and processed using Compass Data Analysis software. The elemental compositions of the present compounds were determined by measuring the *m*/*z* ratio. The proposed molecule for each formula was determined using the KNApSAcK database (last update 2020/09/15).

### 4.4. Cell Lines and Culture

The cervical cancer cell lines used throughout this work are described in Table 8. Because additional cell lines were used only in specific experiments, they are listed in Table S1 (Supplementary Materials). Cell line authentication was performed by short tandem repeat (STR) DNA typing in the Center for Molecular Diagnostics of Barretos Cancer Hospital (São Paulo, Brazil), following the manufacturer’s instructions. All cell lines were tested for the presence of mycoplasma using the MycoAlert Mycoplasma Detection Kit (Lonza, Basel, CHE), according to manufacturer instructions.

### 4.5. Treatments and Cell Viability Assay

Cells were disaggregated by trypsin, counted, and plated at a density of 3 × 10^3^ to 5 × 10^3^ cells per well in a 96-well culture plate. The next day, 10% fetal bovine serum (FBS) medium was replaced with 0.5% FBS medium. After approximately 24 h, the cells were treated with 100 μL medium plus the diluted drugs. Control wells received medium + DMSO (1%). Seventy-two hours after treatment, cell viability was evaluated by a colorimetric assay based on mitochondrial metabolism, Cell Titer 96 Aqueous cell proliferation assay (MTS assay; Promega, Madison, WI, USA) following the manufacturer’s instructions. The absorbance values at 490 nm were read in a microplate reader Varioskan (Thermo Fisher Scientific, Waltham, MA, USA). The half-maximal inhibitory concentration (IC_50_) value was determined by nonlinear regression curve using GraphPad Prism version 5.01 for Windows (GraphPad Software).

The SI was determined to evaluate whether the compounds selectively inhibit the viability of cancer cells versus normal cells [47]. This index was calculated for *A. crassiflora*, *S. adstringens*, and cisplatin. The cisplatin IC_50_ value was determined and those extracts that had at least a two-fold greater effect were considered selective.

The CCC lines were treated with cisplatin to compare the effect of these compounds with treatment used in clinical practice (from 3–250 µg/mL). The stock solution of cisplatin (CIS, Sigma-Aldrich, St. Louis, MO, USA) was diluted in an aqueous solution of 0.9% NaCl and stored at 4 °C.

### 4.6. Analysis of Proteins Involved in Signaling Pathways

To analyze whether *A. crassiflora* fraction 7C24 could modulate signaling pathways involved in cell death, DNA damage, and the cell cycle, we evaluated the expression of cleaved-PARP, phosphor-H2AX, and p21 by Western blotting. Cell lines were selected according to their IC_50_ values, HeLa (15.96 µg/mL) as a low example and SiHa (33.88 µg/mL) as a high one. Cells were seeded into 6-well plates at a density of 0.5 × 10^6^ and treated with an IC_50_ dose of 7C24. After 24 h, the cells were rinsed in Dulbecco’s phosphate-buffered saline (DPBS; Thermo Scientific, Waltham, MA, USA), lysed (50 mM Tris pH 7.6–8, 150 mM NaCl, 5 mM EDTA, 1 mM Na3VO4, 10 mM NaF, 10 mM sodium pyrophosphate, 1% NP-40 and protease cocktail inhibitors), and the sample was scraped and prepared for Western blotting. Proteins were separated by SDS-PAGE gel electrophoresis, transferred to a nitrocellulose membrane, and incubated overnight with primary antibodies targeting PARP, phospho-H2AX, p21 (dilution 1:1000) or α-tubulin (dilution 1:2000), used as the loading control. All antibodies were from Cell Signaling Technology (Danvers, MA, USA). The membranes were incubated with horseradish peroxidase (HRP)-conjugated secondary antibodies (1:5000) and enhanced chemiluminescence Western Blotting Detection Reagents (ECL; RPN2109, GE Healthcare Life Sciences, Pittsburgh, PA, USA), with detection using an ImageQuant LAS 4000 mini documentation system (GE Healthcare Life Sciences, Pittsburgh, PA, USA).

### 4.7. Flow Cytometry

The SiHa cell line was cultured in 6-well plates and treated with 33.88 µg/mL 7C24 (IC_50_ value) for 24 h. The cells were harvested, centrifuged, washed in PBS, and stained with the nuclear marker propidium iodide with the kit BD Cycle test Plus DNA kit (BD Biosciences). Data acquisition was performed using the BD Accuri flow cytometer (BD Biosciences) and was analyzed using the BD Accuri program (BD Biosciences).

### 4.8. Statistical Analysis

An unpaired t-test was used to compare flow cytometry results for the control and treated groups, with *p* < 0.05 considered significant, using the software GraphPad Prism version 5.01 for Windows (GraphPad Software, San Diego, CA, USA, www.graphpad.com, accessed on 1 February 2021).

## Figures and Tables

**Table 1:** IC_50_ values (µg/mL) ^1^ of all crude extracts in human cancer cell lines.

Crude Extracts or Chemotherapeutic	CaSki	SiHa	HeLa
*Tapirira guianensis* (1)	75.39 ± 46.94	121.90 ± 20.08	42.63 ± 13.33
*Astronium fraxinifolium* (2)	168.70 ± 34.67	103.10 ± 48.13	105.30 ± 9.36
*Xylopia aromatica* (3)	84.77 ± 12.78	92.83 ± 30.75	33.34 ± 2.04
*Annona crassiflora* (7)	8.24 ± 1.08	42.44 ± 3.62	18.73 ± 1.80
*Siparuna guianensis* (8)	>300	>300	120.7 ± 9.97
*Achyrocline alata* (10)	71.75 ± 11.53	115.90 ± 39.19	47.85 ± 14.26
*Bauhinia variegata* (14-I)	93.16 ± 16.89	132.80 ± 50.25	39.31 ± 1.56
*Bauhinia variegata candida* (15-I)	111.20 ± 10.39	165.40 ± 60.67	63.36 ± 3.95
*Bauhinia ungulata* (16-I)	169.50 ± 19.37	45.40 ± 0.00	108.70 ± 31.87
*Miconia cuspidata* (17-I)	77.55 ± 14.92	108.80 ± 5.80	91.95 ± 13.51
*Miconia albicans* (18)	68.44 ± 19.31	116.10 ± 0.92	26.95 ± 4.88
*Miconia chamissois* (19)	46.15 ± 8.37	109.60 ± 1.34	11.01 ± 0.84
*Stryphnodendron adstringens* (21-I)	21.12 ± 4.49	47.01 ± 15.78	31.93 ± 4.42
Cisplatin	2.21 ± 0.42	15.48 ± 0.74	6.70 ± 0.43

^1^ Values represent mean ± S.D.

**Table 2:** Selectivity index values of two crude extracts in human cell lines.

Crude Extracts	Selectivity Index		
	CaSki	SiHa	HeLa
*Annona crassiflora*	7.86	1.53	3.46
*Stryphnodendron adstringens*	4.36	1.96	2.88
Cisplatin	2.08	0.29	0.69

**Table 3:** IC_50_ values (µg/mL) ^1^ of *A. crassiflora* partitions in human cervical cancer cell lines.

Partition	Uterine Cervix Cancer Cell Lines		
	CaSki	SiHa	HeLa
7A	12.17 ± 2.32	>50.00	30.99 ± 3.89
7C	15.56 ± 1.85	28.54 ± 4.81	9.45 ± 2.25
7D	7.03 ± 0.43	37.21 ± 16.41	17.11 ± 0.12

^1^ Values represent mean ± S.D.

**Table 4:** Proposed structures by Electrospray Ionization Fourier Transform Ion Cyclotron Resonance Mass Spectrometry (ESI (-) FT-ICR MS) for the identified molecules of chloroform partition of *A. crassiflora*.

*m*/*z* Measured	*m*/*z* Theoretical	Error (ppm)	DBE	[M-H]^−^	Proposed Compound	Reference
255.23315	255.23295	−0.75	1	[C_16_H_32_O_2_− H+]−	Hexadecanoic acid (palmitic acid)	[20]
281.24884	281.24860	−0.85	2	[C_18_H_34_O_2_− H+]−	(Z)-9-Octadecenoic acid (oleic acid)	KNApSAcK database; [20]
297.24379	297.24352	−0.92	2	[C_18_H_34_O_3_− H+]−	Ricinoleic acid	KNApSAcK database; [20]
353.08821	353.08781	−1.13	8	[C_16_H_18_O_9_− H+]−	4-O-E-caffeoylquinic acid	[21]
415.12514	415.12459	−1.35	7	[C_18_H_24_O_11_− H+]−	Alpinoside	KNApSAcK database
463.08891	463.08820	−1.53	12	[C_21_H20O_12_− H+]-	Quercetin 3-O-glucoside	[22]
477.10455	477.10385	−1.46	12	[C_22_H_22_O_12_− H+]−	Quercetin-*O*-methyl-*O*-hexoside	[21]
281.24879	281.24860	−0.66	2	[C_18_H_34_O_2_− H+]−	(Z)-9-Octadecenoic acid (oleic acid)	KNApSAcK database; [20]
431.09868	431.09837	−0.72	12	[C_21_H20O_10_− H+]−	kaempferol-3-O-rhamnoside	[23,24]

*m*/*z* (mass-to-charge ratio); ppm (parts per million); DBE (double bond equivalent).

**Table 5:** IC_50_ values ^1^ of *A. crassiflora* fractions in cervical cancer cells.

Partition	IC_50_ (µg/mL)			
	Uterine Cervix Cancer Cell Lines			
	CaSki	SiHa	HeLa	C4-I
7C18	-	>50.00	>50.00	-
7C22	-	>50.00	>50.00	-
7C24	34.8 ± 3.67	33.88 ± 4.17	15.96 ± 0.89	15.01 ± 5.20
7C25	41.14 ± 6.86	>50.00	35.55 ± 1.06	23.9 ± 8.74
7C28	29.13 ± 8.34	35.85 ± 8.29	27.7 ± 2.86	13.9 ± 5.16
7C39	-	>50.00	>50.00	-
7C45	>50.00	>50.00	39.64 ± 0.86	31.95 ± 10.66
7C52	-	>50.00	>50.00	-
7C57	>50.00	>50.00	32.58 ± 0.69	14.30 ± 6.76
7C60	32.11 ± 8.90	>50.00	23.47 ± 10.56	17.40 ± 4.13

^1^ Values represent mean ± S.D.—(Not tested).

**Table 6:** Proposed structures for components of the 7C24 fraction of *A. crassiflora*.

*m*/*z* Measured	*m*/*z* Theoretical	Error (ppm)	DBE	[M-H]^−^	Proposed Compound	Reference
281.24879	281.24860	−0.66	2	[C_18_H_34_O_2_− H+]−	(Z)-9-Octadecenoic acid (oleic acid)	KNApSAcK database; [20]
431.09868	431.09837	−0.72	12	[C_21_H20O_10_− H+]−	kaempferol-3-O-rhamnoside	[23,24]

*m*/*z* (mass-to-charge ratio); ppm (parts per million); DBE (double bond equivalent).

**Table 7:** Characteristics of the Cerrado plant species.

Crude Extracts Identification	Vernacular Name	Scientific Name	Family	Register
1	Pau pombo, Carvoeiro, Arapaçu, Carvoeiro do Cerrado	*Tapirira guianensis*	Fabaceae	143407 BHCB
2	Gonçalo-Alves	*Astronium fraxinifolium*	Anacardiaceae	143403 BHCB
3	Pimenta-de-Macaco	*Xylopia aromatica*	Annonaceae	43397 BHCB
7	Araticum, ariticum	*Annona crassiflora*	Annonaceae	143400 BHCB
8	Negramina, Caapitiú,Catingueira-de-paca, Erva-de-rato	*Siparuna guianensis*	Siparunaceae	143404 BHCB
10	Jatei-kaá	*Achyrocline alata*	Asteraceae	11486 CGMS
14-I	Pata-de-vaca ou casco-de-vaca lilás	*Bauhinia variegata*	Fabaceae	161589 BHCB
15-I	Pata-de-vaca branca ou casco-de-vaca branco	*Bauhinia variegata candida*	Fabaceae	161590 BHCB
16-I	Pata-de-viado	*Bauhinia ungulata*	Fabaceae	161588 BHCB
17	Pixirica-da-mata	*Miconia cuspidata*	Melastomataceae	44998 HUFU
18	Canela de velho	*Miconia albicans*	Melastomataceae	56558 HUFU
19	Pixirica-açu, Roxinha do brejo, Fruta de Chupim do brejo, Folha larga do brejo e folha de bolo	*Miconia chamissois*	Melastomataceae	59592 HUFU
21-I	Barbatimão	*Stryphnodendron adstringens*	Fabaceae	169871 BHCB

BHCB (Herbarium of the Federal University of Minas Gerais); CGMS (Herbarium of the Federal University of Mato Grosso do Sul, Campo Grande campus, Brazil); HUFU (Herbarium of the Federal University of Uberlândia).

**Table 8:** Cancer cell lines in the main panel.

	Cell Line	Tissue	Histologic Type	Gender	Age	Supplier	Culture Medium
1	CaSki *	Uterine cervix	Squamous cell carcinoma	Female	40	ATCC	DMEM
2	SiHa *	Uterine cervix	Squamous cell carcinoma	Female	55	ATCC	DMEM
3	HeLa	Uterine cervix	Adenocarcinoma	Female	31	ATCC	DMEM

Media supplemented with 1% Penicillin/Streptomycin and 10% fetal bovine serum (FBS). ATCC (American Type Culture Collection). DMEM (Dulbecco’s modified Eagle’s medium) * Kindly provided by Dra. Luisa Lina Villa.

## Data Availability

The data presented in this study are available upon request from the corresponding author.

## Supplementary Materials

The following are available online at https://www.mdpi.com/1422-0067/22/7/3383/s1, Table S1: Cell lines, Table S2: IC_50_ values (µg/mL) of all crude extracts in human cell lines.
