# Supplementary material for: Bioprospecting of Natural Compounds from Brazilian Cerrado Biome Plants in Human Cervical Cancer Cell Lines
Source: Int J Mol Sci. 2021 Mar 25;22(7):3383. doi: 10.3390/ijms22073383 (PMC8036847; doi:10.3390/ijms22073383)
Supplement: Supplementary file 1 [file ijms-22-03383-s001.pdf]

International Journal of *Molecular Sciences*

MDPI

Supplementary Materials

Article

Bioprospecting of natural compounds from Brazilian Cerrado biome plants in human cervical cancer cell lines

Marcela N. Rosa[1], Larissa R. V. e Silva[1], Giovanna B. Longato[1,2], Adriane F. Evangelista[1], Izabela N. F. Gomes[1], Ana Laura V. Alves[1], Bruno G. de Oliveira[3], Fernanda E. Pinto[3], Wanderson Romão[3], Allisson R. de Rezende[4], Arali A. C. Araújo[4], Lohanna S. F. M. Oliveira[5], Alessandra A. de M. Souza[5], Stephanie C. Oliveira[5], Rosy Iara M. de A. Ribeiro[5], Viviane A. O. Silva[1], Rui M. Reis[1,6,7*]

1 Molecular Oncology Research Center, Barretos Cancer Hospital, Barretos, São Paulo, Brazil; nr.marcela2@gmail.com (M.N.R.); larirussoveloso@yahoo.com.br (L.R.V.S.); adriane.feijo@gmail.com (A.F.E.); izabela.faria.tk@hotmail.com (I.N.F.G.); alves.anav@gmail.com (A.L.V.A.); vivianeaos@gmail.com (V.A.O.S)

2 Research Laboratory in Molecular Pharmacology and Bioactive Compounds, São Francisco University, Bragança Paulista, São Paulo, Brazil; giovanna.longato@usf.edu.br (G.B.L.)

3 Petroleomic and Forensic Laboratory, Chemistry Department, Federal University of Espírito Santo, Vitória, ES, Brazil; brunoliveir_ra20@msn.com (B.G.O); fernandapinto80@gmail.com (F.E.P.); wandersonromao@gmail.com (W.R.)

4 University of the state of Minas Gerais (UEMG), Ituiutaba, MG, Brazil; rodrigues.allisson@gmail.com (A.R.R.); arali.dacov@gmail.com (A.A.C.A.)

5 Laboratory of Experimental Pathology, Federal University of São João del Rei—CCO/UFSJ, Divinópolis, Brazil; lohh.franca@gmail.com (L.S.F.M.O.); aleapms@gmail.com (A.A.M.S.); rosy@ufsj.edu.br (R.I.M.A.R.)

6 Life and Health Sciences Research Institute (ICVS), School of Medicine, University of Minho, Braga, Portugal;

7 ICVS/3B's- PT Government Associate Laboratory, Braga/Guimarães, Portugal;

* Correspondence: ruireis.hcb@gmail.com; Tel.: +55 173 321 6600;

International Journal of *Molecular Sciences*

MDPI

Table S1 (Supplementary Materials) Cell lines.

	Cell line	Tissue	Histologic type	Gender	Age	Supplier	Culture medium*
1	HaCat[#]	Skin	Keratinocyte	Male	62	ATCC	DMEM
2	C33A[#]	Uterine cervix	Squamous cell carcinoma	Female	66	ATCC	DMEM
3	HtTA-1	Uterine cervix	Adenocarcinoma	Female	31	ECACC	DMEM
4	HR5	Uterine cervix	Adenocarcinoma	Female	31	ECACC	DMEM
5	HR5-CL11	Uterine cervix	Adenocarcinoma	Female	31	ECACC	DMEM
6	Bu25TK	Uterine cervix	Adenocarcinoma	Female	31	ECACC	DMEM
7	C4-I	Uterine cervix	Squamous cell carcinoma	Female	41	ECACC	DMEM

*Medium was supplemented with 1% Penicillin/Streptomycin and 10% fetal bovine serum (FBS). ATCC (American Type Culture Collection). ECACC (European Collection of Authenticated Cell Cultures). [#]Kindly provided by Dra. Luisa Lina Villa.

Table S2 (Supplementary Materials) IC_{50} values (μg/mL)[a] of all crude extracts in human cell lines.

**Crude extracts or chemotherapeutic**	**Uterine cervix cancer cells**					**Normal cells**
	**HR5**	**HR5-CL11**	**HtTA-1**	**Bu25TK**	**C33**	**HaCat**
Tapirira guianensis (1)	49.25 ± 13.38	37.19 ± 21.66	27.65 ± 11.30	48.91 ± 1.31	-	-
Astronium fraxinifolium (2)	133.50 ± 36.84	82.54 ± 14.96	90.17 ± 6.94	108.40 ± 5.44	-	-
Xylopia aromatica (3)	38.67 ± 11.91	39.78 ± 8.41	13.81 ± 11.44	29.23 ± 10.72	-	-
Annona crassiflora (7)	14.60 ± 1.63	15.36 ± 1.92	2.70 ± 0.99	13.97 ± 0.04	-	64.73 ± 5.9
Siparuna guianensis (8)	-	-	-	-	37.91 ± 15.80	-
Achyrocline alata (10)	-	-	24.65 ± 13.55	-	-	-
Bauhinia variegata (14-I)	-	-	50.36 ± 9.17	-	-	-
Bauhinia variegata candida (15-I)	-	-	73.52 ± 7.86	-	-	-
Bauhinia ungulata (16-I)	-	-	153.50 ± 35.85	-	-	-
Miconia cuspidata (17)	-	-	-	-	66.80 ± 0.00	
Miconia albicans (18)	-	-	-	-	40.65 ± 5.06	
Miconia chamissois (19)	-	-	-	-	33.53 ± 0.13	
Stryphnodendron adstringens (21-I)	-	-	25.09 ± 11.38	-	-	92.07 ± 2.35
Cisplatin	N.D.	1.98 ± 0.03	18.55 ± 3.60	12.3 ± 0.99	-	4.6 ± 1.48

[a] Values represent mean ± S.D. - (Not tested).
